# Spoilage Lactic Acid Bacteria in the Brewing Industry

**DOI:** 10.4014/jmb.1908.08069

**Published:** 2020-01-09

**Authors:** Zhenbo Xu, Yuting Luo, Yuzhu Mao, Ruixin Peng, Jinxuan Chen, Thanapop Soteyome, Caiying Bai, Ling Chen, Yi Liang, Jianyu Su, Kan Wang, Junyan Liu, Birthe V. Kjellerup

**Affiliations:** 1 School of Food Science and Engineering, Guangdong Province Key Laboratory for Green Processing of Natural Products and Product Safety, South China University of Technology, Guangzhou 510640, P.R. China; 2 Department of Microbial Pathogenesis, School of Dentistry, University of Maryland, Baltimore, MD 21201, USA; 3 Home Economics Technology, Rajamangala University of Technology Phra Nakhon, Bangkok, Thailand; 4 Guangdong Zhongqing Font Biochemical Science and Technology Co. Ltd., Maoming, Guangdong 525427, P.R. China; 5 Research Center of Translational Medicine, Second Affiliated Hospital of Shantou University Medical College, Shantou 515041, Guangdong, P.R. China; 6 Guangdong Women and Children Hospital, Guangzhou 510010, P.R. China; 7 Department of Civil and Environmental Engineering, University of Maryland, College Park, MD 20742, USA

**Keywords:** Lactic acid bacteria, brewing industry, spoilage mechanism, detection methods

## Abstract

Lactic acid bacteria (LAB) have caused many microbiological incidents in the brewing industry, resulting in severe economic loss. Meanwhile, traditional culturing method for detecting LAB are time-consuming for brewers. The present review introduces LAB as spoilage microbes in daily life, with focus on LAB in the brewing industry, targeting at the spoilage mechanism of LAB in brewing industry including the special metabolisms, the exist of the viable but nonculturable (VBNC) state and the hop resistance. At the same time, this review compares the traditional and novel rapid detection methods for these microorganisms which may provide innovative control and detection strategies for preventing alcoholic beverage spoilage, such as improvement of microbiological quality control using advanced culture media or different isothermal amplification methods.

## Introduction

Beer is considered one of the most common beverages with high microbiological stability due to the existence of hop bitter acids, alcohol, and low content of oxygen and nutrients. However, a few microbes can still grow in beer with the turbidity and acidity, even producing pathogenic chemicals [1]. Among all the harmful microorganisms in the brewing industry, lactic acid bacteria contribute to almost 70% of all safety-related incidents. These spoilers can survive in these unfavorable conditions causing false-negative detection due to their hop resistance and ability to enter a viable but nonculturable (VBNC) state under stress conditions [2, 3].

## Lactic Acid Bacteria as Food Spoiler

Lactic acid bacteria (LAB) are facultative anaerobes, and because they utilize fermented carbon hydrate to produce lactic acid, they are useful in producing yogurt and pickles [4]. LAB can be divided into at least 18 genera, with more than 200 species. But only a few of them have the ability to cause spoilage of food. *Lactobacillus* and *Pediococcus* are recognized as the most hazardous bacteria in the brewing industry since they are responsible for nearly 70% of the microbial beer-spoilage events. They cause turbidity and buttery odor and sourness in alcoholic beverages mainly due to the formation of diacetic acid, lactic acid and extracellular polysaccharide which render beverages undrinkable [5].

In the wine industry, *Pediococcus* produce lactic acid through malolactic fermentation. Their secondary metabolites are mainly volatile substances, so if they are rapidly propagated in the wrong time period, they will affect the flavor of the wine and become spoilage microorganisms. Also, their extracellular polysaccharide contributes to an increase in viscosity [5-7]. Currently, there are 15 species of *Pediococcus*, of which only *P. damnosus* can survive in conditions above 10% NaCl and low pH (4.5) [8]. Among the components of beer, hop compounds protect beer from spoilage by most bacteria. Most of the bacteria are inhibited by the low acidity of the beer and the antiseptic effect of the hops, but *P. damnosus*, *Lb. brevis, Lb. lindneri* have anti-hop properties and can adapt to the beer environment, resulting in prolonged fermentation time and high levels of diacetyl accumulation, causing the beer to have a butter odor or sour taste accompanied by sticky silk and turbidity [9]. Therefore, studying the resistance mechanism of LAB to hops is significant for understanding their spoilage ability.

Other than beer, lactic acid bacteria are also commonly found in vacuum packaging and modified atmosphere packaging for meat products where they are known as dominant spoilage flora under anaerobic conditions. They are resistant to the bacteriostatic action of nitrite and smoke in processed meat products, while at the same time being able to tolerate higher concentrations of salt. LAB can use the carbohydrates in the fermented meat to produce sour taste, cheese flavor and the smell of the animal liver, which is sometimes accompanied by the production of carbon dioxide. According to the current reports, meat-related lactic acid bacteria are *Lactobacillus, Lactococcus, Leuconostoc, Carnivora, and Weissella* [10]. Among of them, *Lactobacillus* spp*.* are the most common spoilage bacteria in packaged meats with characteristics of sour taste due to the production of lactic acid, acetic acid and formic acid. Also, this off-odor is related to the formation of acetylene vinyl and 3-methylbutanol [11]. Studies have shown that *Lb. sakei, Lb. curvatus, Lb. algidus, Lb. fuchuensis, Lb. oligofermentans* are related to the decay of poultry, cured meat and ground pork in vacuum packaging and modified atmosphere packaging with unpleasant smell, acidification and the presence of mucus [12, 13]. Researchers determined that *Lb. sakei* was isolated from low-acid fermented sausages and are dominant spoilage bacteria in vacuum packaging processing of meat products, capable of producing greenish coloration caused by hydrogen sulfide, resulting in muscle pigmentation converted to green vulgaris myoglobin [11, 14]. Formation of organic acid by *Leu. gelidum, Leu.carnosum* and *Leu.mesenteroides* also causes “cheese flavor”, forming of mucus, production of gas and greening in some meat products [10, 15].

Spoilage induced by LAB is also common in dairy products like milk. LAB contribute flavor defects, “sour” off-flavors by producing acetic and propionic acids as a by-product of metabolic reactions [5]. In the latehere was high incidence of raw milk spoilage with malty flavor contributed by *Lc. lactis*. Also, in 1947, researchers concluded that various aldehydes and alcohols such as 2-methylbutanal and 3-methylbutanal create a “malty flavor” in milk by *Lc. lactis* [16]. Milk spoilage caused by LAB is due to inappropriate storage conditions, especially relating to the temperature. It has been demonstrated that the relative proportions of *Lc. Lactis,* which are the major raw milk bacterial component, decrease after storage at 4°C for 24 h [17]. The phenomenon of cheese spoilage is also related to lactic acid bacteria. Deterioration of cheese produces unpleasant odors, gases and the formation of white calcium lactate crystals on the surface which are contributed by nonstarter lactic acid bacteria (NSLAB). The surface of the device for producing a stirred curd-type cheddar cheese can coated with an erythrocyte-resistant NSLAB *(Lb. curvatus* or *Lb. fermentum*) biofilm. During processing, the biofilm can peel off and contaminate the product, so the formation of the biofilm allows the bacteria to survive the sterilization process and eventually leads to deterioration of the cheese [18]. *Lactobacillus spp*. also causes spoilage of other products. Ulrike Lyhs *et al.* found that *Lb. alimentarius* was the dominant gas-borne spoilage causing the defect of pickled herring [19]. Unlike "carbohydrate swell," it causes a slight increase in the pH of canned fish products and they refer to this form of decay as "protein swell". Researchers even determined that *Lb. acetotolerans* was able to survive in an environment with acetic acid concentrations of 4% and cause vinegar spoilage [20].

Table 1 summarizes spoilage LAB in different foods in the current review to provide a comprehensive overview of these microbes spoilage LAB and the typical characteristics of defect.

## Spoilage Mechanisms of LAB in the Brewing Industry

### Metabolism

LAB are the predominate spoilage microorganisms in the brewing industry, causing not only off-flavor but also altering the viscosity and turbidity of beer. There is no significant correlation between the metabolic capacity of lactic acid bacteria and the potential for spoilage. However, the metabolism of fermentation type could reveal that the spoilage is mainly caused by differences in amino acid and carbohydrate metabolism [21]. According to the metabolic activity of LAB, they can be classified into three types: obligately homofermentative, obligately heterofermentative and facultatively heterofermentative. Homofermentative LAB produce D-lactic acid while heterofermentative LAB produce D-lactic acid and acetic acid which are associated with spoilage due to the increase of acid content in the product. Acetic acid is perceived as vinegar and lactic acid as stale milk and yoghurt-like [5]. Some compounds, such as diketone, 2-3-butanedione or diacetyl produced from citrate metabolism, are perceived as the major source of pleasant flavor at low concentrations, while at high concentrations they are considered to be a spoilage characteristic [22]. Mannitol, which is produced by heterofermentative LAB (*Lb. brevis and Lb. lindneri*), is not responsible for spoilage[21], but when it works together with acetic acid, D-lactic acid, n-propanol, 2-butanol, and diacetyl, beer shows spoilage characteristics with viscous texture, sweet taste and vinegar-estery aroma [23, 24]. Moreover, LAB are regarded as the main biogenic amine (BA) producers in fermented food due to the conversion of available amino acid precursors to BAs via activities of decarboxylase or deiminase. These compounds are toxic chemicals causing negative symptoms, such as headache, vomiting and diarrhea. Heterofermentative *Lb. brevis* and *Lb. lindneri* from spoilage beer accumulate tyramine and histamine, respectively, which are the most dangerous BAs affecting the hygienic quality of beer [25].

### VBNC State

In 1982, an investigator found a special state for bacteria which was designated viable but non-culturable’ (VBNC) or viable Putative but Nonculturable (VPNC) with the reduction of cells, decrease of metabolic activity and the change of membrane composition and cell wall structure [26, 27]. This special survival strategy of bacteria can be induced by many factors, such as starvation condition, adverse temperature and even UV disinfectants [28, 29]. Under this special state, bacteria remain alive with minimal level of metabolic activity but fail to be cultured by routine bacteriological media resulting in the false-negative identification of VBNC bacteria. However, bacteria in a VBNC state can be resuscitated and revert to the normal state once given the appropriate conditions, including upshift of temperature and addition of catalase which may provide guidance on prevention and control of beer spoilage [30]. The VBNC state is regarded as a common strategy to survive in both gram-positive and gram-negative bacteria. Hence, the VBNC pathogenic bacteria are considered to be an important threat.

In 2006, researchers induced beer spoilers, *Lb. lindneri* as well as *Lb. acetotolerans*, into VBNC state successfully by cold treatment and beer subculture treatment which determined that VBNC beer spoilage LAB can be ignored easily in the brewing industry [31, 32]. A later study investigated the beer spoilage capability of VBNC *Lb. lindneri* by reversed-phase high-performance liquid chromatography and head space gas chromatography. Compared with normal state, beer with VBNC and resuscitated cells showed a slight increase in lactic acid and acetic acid after being incubated for one month [33]. For *Lb. plantarum,* there was no significant difference in spoilage ability between normal and VBNC cells. However, *Lb. plantarum* and *Lb. acetotolerans* VBNC cells did cause turbidity in beer after one month [30, 34]. Results were similar in the study of beer-spoilage capacity of *Lb. brevis* BM-LB13908 [35].

Further study analyzed the genomic of *Lb. casei* BM-LC14617, which is beer-spoilage bacteria with several potential VBNC genes identified by Gene Ontology (GO) functional, Clusters of Orthologous Groups (COG) functional and Kyoto Encyclopedia of Genes and Genomes (KEGG) pathway annotations. These predicted genes were associated with defense and stress response, including oxidative stress, antibiotics resistance and metal resistance [36]. The sigma factor encoded by *rpoS* gene was suggested to be required for survival of bacteria under diverse environmental stresses [37]. In a recent report, researchers confirmed the same result that the sigma factor was associated with VBNC state. The transcriptional of *rpoS* was related to the production of guanosine 3’,5’-bispyrophosphate (ppGpp) [38]. However, the absence of this gene in the genome of *Lb. acetotolerans* BM-LA14527 and *Lb. harbinensis* BM-LH14723 indicated that the sigma factor RpoD and other sigma factors might be alternative sigma factors associated with the formation of VBNC state [39, 40]. Functional and pathway enrichment analysis also revealed that response of adverse environmental conditions and the down-regulation of gene expression involving metabolic processes are significant for the VBNC state [41].

At present, research on the status of VBNC in the susceptible *Lactobacillus* beer is still in its infancy and should receive more attention.

### Hop Resistance

Beer has been recognized as a beverage with high microbiological stability. But some spoilage *Lactobacillus* are able to grow in beer and cause defect. The iso-α-acids from the hop exert an antibacterial effect on gram-positive bacteria including most LAB due to their ability to dissipate the proton motive force. However, there are some exceptions which are able to grow in hopped beer, such as *Lactobacillus* spp. The hop resistance was reported to be associated with *horA* and *horC* genes [42, 43]. A researcher discovered that plasmid pRH45, which harbors *horA* gene confers hop resistance to cell. The amino acid sequence derived from the *horA* gene is 53% identical to the sequence of the multidrug transporter LmrA of *Lc. lactis* and has ability to transport various amphiphilic compounds. The expression of *horA* enhances the hop resistance of *Lc. lactis*. The multi-drug transporter encoded by the *horA* gene is capable of transporting hops from cells, thereby protecting LAB from hop compounds [44]. *HorA* was reported to be an ATP-dependent multidrug transporter. According to the results of Suzuki *et al.* [45], three beer-spoilage *Lactobacillus* strains exhibited strong ATP-yielding ability by consuming citrate, pyruvate, malate, arginine and sometimes even maltotriose. Xu *et al.* [46, 47] performed a draft genome study on *Lb. acetotolerans BM-LA14527* combined with GO function annotations, COG functional annotations and KEGG biological pathway annotations of predicted genes, and obtained the same results.

However, *Lactobacillus* lacking *horA* gene is still able to grow in beer, although its growth ability is weak. This demonstrates that there are other factors that make *Lactobacillus* resistant to hops. Later, another study found the open reading frame (ORF) associated with hop resistance. *HorB* and *horC* were renamed from ORF1 and ORF2 in *Lb. brevis ABBC45* which have ability to encode a multidrug transporter to confer hop resistance [48]. Also, researchers demonstrated that *Lb. brevis ABBC45cc* with *horC* and its putative regulator *horB* exhibited beer-spoilage ability and higher hop resistance than *Lb. brevis ABBC45cc* with *horB* which demonstrated the fact that *horC* plays a key role in beer-spoilage and hop resistance [49]. Studies have shown that the second mechanism of hop resistance was mediated by proton-motive-force (PMF)-dependent multidrug resistance pump encoded by *horC* [49, 50]. Furthermore, the homologs of *horA* and *horC* genes were widely discovered in various beer-spoilage LAB strains indicating both the genes can potentially be genetic markers to distinguish the spoilage ability [51].

Additionally, ORF5 found in pRH45II was identified. The ORF5 homologue was present in all beer-spoilage *Lactobacillus* in the experiments and not present in non-spoilage mutants indicating it is potentially a useful genetic marker [50, 52].

## Classical and Innovative Detection Methods of Food Spoilage Microorganisms

The traditional detection methods of microorganisms are standard plate counting method and direct microscope observation method. These methods require simple equipment with the cumbersome detection operation, long period and low sensitivity. In the brewing industry, the detection of beer-spoilage LAB using traditional plate culture method is a great challenge owing to the difficulty of growing on the culture medium which requires up to 10 days. However, the addition of catalase in De Man Rogosa Sharpe (MRS) culture medium enables improvement of the growth and colony sizes of beer-spoilage LAB within five days which shortens the detection time comparing with the traditional culture method [53].

Different from traditional media, chromogenic media are simple to operate with little time requirement and high sensitivity and specificity which employ the visible color change of hydrolyzed enzymes completing the detection and identification. These chromogenic media have been utilized to identify several microorganisms including *Bacillus cereus, Enterobacter sakazakii, Listeria monocytogenes* and *Staphylococcus aureus* [54-57]. Compared to the standard media, new chromogenic media are better for isolation and identification of microorganisms accurately. However, some strains still could not be detected by this new chromogenic media due to the specific variances. Thus, this method still has its limitation. Furthermore, the finding of microbes in VBNC state makes these culture media methods highly challenging. However, combined with the acridine orange direct counting, Live/Dead BacLight bacterial viability kit and fluorescence microscope, the VBNC state cells can be detected, although it is a complicated method.

Polymerase chain reaction (PCR) and isothermal amplification are two main assays for amplifying target sequence of nucleic acid to detect and identify microorganisms. PCR has been a conventional method for a long time. Advanced detection techniques with rapid and high sensitivity but bulky operation, including real-time PCR (q-PCR), are widely used for spoilage bacteria identification in the food industry, including spoilage yeasts, *Pseudomonas* and *Clostridia* [58-60]. Moreover, compared with culture media, amplification assays are able to detect VBNC state cells which still possess the conserved genes. Propidium monoazide (PMA) is a photosensitive dye that has a high affinity with DNA. The dye cannot penetrate into the intact cell membrane but only selectively pass through the dead cells damaged by the cell membrane. Therefore, PMA-PCR and PMA-qPCR can be utilized to verify and detect the existence of VBNC cells precisely [58].

Other than PCR assays, there are various isothermal amplification methods to conduct the detection of microorganisms, such as loop-mediated amplification (LAMP), rolling circle amplification (RCA), strand displacement amplification (SDA) and cross-priming amplification (CPA) [62-65]. Compared with PCR, these detection methods do not require frequent and accurate temperature change, but do require multiple enzymes and/or special reagents. Zhang *et al*. designed the primers by using the gene sequence of the conserved region of *Lb. acidophilus* 16sRNA and optimized the LAMP reaction system, making it possible to rapidly detect *Lb. acidophilus* [66]. Researchers also utilized the LAMP assay to detect *P. fluorescens* in milk. Results showed that compared with PCR assay, the detection limit of LAMP reaction is 10-100 times that of PCR [67]. Rapid detection of *Lactobacillus* in beer can be carried out by RCA reaction. Hua Yin *et al*. designed a padlock probe and primers for the beer spoilage *horA* gene in *Lb. acetotolerans*, and successfully carried out amplification reaction at 42.7°C [68]. Generally, for these amplification assays, the amplicons would be detected on gel electrophoresis employing agarose gel which is a time-consuming process. However, by using fluorescence dye, such as SYBR Green, amplification products can be visually detected both by naked eye and under UV light [69, 70]. In this way, detection time can be greatly reduced and the application of on-site rapid detection can be possible. In addition, the isothermal amplification can also combine with PMA to detect the VBNC cells.

Matrix-assisted laser desorption/ionization time-of-flight mass spectrometry (MALDI-TOF MS) is an important advanced technology to rapidly detect bacteria at genus, species and strain levels by using the mass profile of molecular analytes [71, 72]. Seafood-spoilage bacteria including *Pseudomonas, Enterobacter, Serratia* and *Shewannella* can be characterized by MALDI-TOF MS in a fast procedure [73]. In the present study, MALDI-TOF MS was developed to identify the different LAB found in fermented food [74-76]. Besides bacteria, yeasts are able to be identified through this new method. *Bellanger et al*. successfully identified *Candida* spp. from chromogenic Candida medium instead of different methods by MALDI-TOF MS and determined the three technical parameters to promote a successful identification including conditions of incubation, culture media and minimal time point of culture [77]. Table 2 summarizes the novel detection methods of spoilage LAB based on the current study which might give an innovative look into the future detection of LAB.

## Discussion

Food safety has always been a common concern across the world. Food spoilage microorganisms that can contaminate various foods pose a serious threat in daily life. Lactic acid bacteria have long been considered to be beneficial in fermented food, but in fact they can be the spoiler of various foods especially in the brewing industry. Some LAB have ability of hop resistance and produce harmful metabolisms that contribute to beer spoilage. Also, various studies confirmed that LAB is able to exist in a VBNC state causing defects in alcoholic beverages and false-negative results by traditional culture methods. Therefore, it is still necessary to conduct the further systematic studies on gene expression, regulation and transcription in order to determine the specific mechanisms of these microorganisms which may contribute effective measures to control food spoilage caused by microbes.

As for the detection of hazardous LAB in the brewing industry, development of culture methods does bring great convenience to the detection of microorganisms. However, the discovery of VBNC status has become a defect that cannot be detected. Considerable identification and detection assays for food spoilage microbes have been developed successfully. Molecular biology-based nucleic acid amplification technology can detect bacteria in VBNC state including PCR and isothermal amplification. In the future, however, more rapid and advanced detection methods are required to reduce the false-positive results in testing to ensure the accuracy and achieve successful on-the-spot inspection.

## Figures and Tables

**Table 1:** Summary of spoilage LAB.

Food sources	Spoilage LAB	Characteristics of defect	References
Wine	*Pediococcus* spp.	Increased viscosity and slimy appearance	[5-7]
Beer	*Lb. brevis, Lb. lindneri, Pediococcus*spp.	Butter odor or sour taste, sticky silk and turbidity	[3,21,33,35,44]
Packaged meat products	*Lb. sakei, Lb. curvatus, Lb. algidus, Lb. fuchuensis, Lb. oligofermentans Leu. gelidum, Leu. carnosum Leu. mesenteroides*	Off-odor and acidification sometimes with the production of carbon dioxide and hydrogen sulfide and formation of mucus	[10-15]
Milk	*Lc. lactis*	Sour taste	[16,17]
Cheese	*Lb. curvatus, Lb. fermentum*	Unpleasant odors, gases and the formation of white calcium lactate crystals	[18]
Canned fish products	*Lb. alimentarius*	Protein swell and the formation of gas	[19]
Vinegar	*Lb. acetotolerans*	Turbidity	[20]

**Table 2:** Summary of the novel detection methods of spoilage LAB.

Spoilage LAB	Detection method	Detection time	References
*Lb. brevis, Lb. plantarum, Lb. acetotolerans,*	De Man Rogosa Sharpe (MRS) culture	2-5 days	[53]
*P. damnosus*	Medium with catalase added		
*Lb. acidophilus*	LAMP^a^	5.5 h	[66]
*Lb. acetotolerans*	RCA^b^	2.5h	[68]
*Lb. fermentum, Lb. brevis, Lb. plantarum,*	MALDI-TOF MS^c^	ND	[74-76]
*Lactococcus spp., Leuconostoc* spp*.*			

a LAMP = loop-mediated isothermal amplification

b RCA = rolling circle amplification

c MALDI-TOF MS = matrix-assisted laser desorption/ionization time-of-flight mass spectrometry ND=not defined
